# Relatives’ Involvement in the Care Pathway of Patients With Acquired Brain Injury or Malignant Brain Tumour: An Observational Study

**DOI:** 10.1155/nrp/2280006

**Published:** 2026-04-19

**Authors:** Rikke Guldager, Emilie Gyldenöhr, Ingrid Poulsen, Lena Aadal, Sara Nordentoft, Mia Ingerslev Loft

**Affiliations:** ^1^ Department of Neurosurgery, The Neuroscience Centre, Copenhagen University Hospital – Rigshospitalet, Copenhagen, Denmark; ^2^ Department of People and Technology, Roskilde University, Roskilde, Denmark, ruc.dk; ^3^ Department of Clinical Research, Copenhagen University Hospital–Amager and Hvidovre Hospital, Hvidovre, Denmark; ^4^ Hammel Neurorehabilitation and Research Centre, Hammel, Denmark; ^5^ Department of Clinical Medicine, Aarhus University, Aarhus, Denmark, au.dk; ^6^ Department of Neurology, The Neuroscience Centre, Copenhagen University Hospital–Rigshospitalet, Copenhagen, Denmark; ^7^ Department of Clinical Medicine, University of Copenhagen, Copenhagen, Denmark, ku.dk

**Keywords:** acquired brain injury, communication, family-centred care, malignant brain tumour, neurological nursing, nonparticipant observation, qualitative content analysis, relative involvement

## Abstract

**Aim:**

To explore how nurses involve the relatives of patients with acquired brain injury or malignant brain tumour and how this involvement unfolds in hospital ward settings. We focus on the contextual, interpersonal, and communicative dynamics that shape and mediate involvement.

**Design:**

A qualitative, exploratory design using nonparticipant field observation.

**Methods:**

Nineteen nonparticipant observations were conducted across seven units in four Danish hospital sites, covering the full clinical pathway from acute care to inpatient rehabilitation. Data were analysed using inductive content analysis following Graneheim and Lundman approach.

**Results:**

The analysis identified one overarching theme and three categories that illuminate how relative involvement is shaped in clinical practice. Relatives’ opportunities to be involved in the patient’s treatment and care were strongly influenced by the physical and organizational context, including the spatial layout of wards and the timing of clinical routines. Communicative practices ranged from one‐way information delivery to inclusive dialogues, affecting whether relatives were acknowledged and involved. The way nurses recognized relatives relationally, either as passive bystanders or as knowledgeable contributors, further shaped their role. Across all categories, a continuum of involvement emerged, ranging from complete absence to active partnership, reflecting how structural and interpersonal factors interact to enable or hinder meaningful engagement.

**Conclusion:**

Relative involvement is not consistently embedded in everyday clinical routines but varies depending on context, professional practice, and communicative approach. Involvement often depends on relatives taking initiative and is rarely proactively facilitated by nurses. However, when nurses are able to create structured, inclusive environments, relatives shift from peripheral observers to valued partners in care.

**Impact:**

This study contributes important observational knowledge to the field of family involvement in acute and complex care settings. The findings highlight the need for structured, context‐sensitive strategies that enable nurses to identify and support diverse forms of relative involvement. Such efforts are vital to ensure equitable engagement across different clinical settings and family constellations.

## 1. Background

Acquired brain injury (ABI) and malignant brain tumour (MBT) are serious neurological conditions that typically result in complex and multifaceted impairments affecting multiple domains of patients’ lives. ABI may arise from traumatic events, hypoxia, haemorrhage, infection, or other nondegenerative causes and often leads to cognitive deficits (e.g., impaired memory, attention, or executive functioning), physical impairments such as motor weakness or reduced mobility, and psychological or behavioural changes including irritability, emotional instability, and reduced insight [1–4]. Similarly, MBT is associated with tumour‐related neurological symptoms such as seizures, headaches, focal deficits, fatigue, and progressive cognitive decline which may develop rapidly depending on tumour type and growth dynamics [4–6]. Together, these impairments substantially limit patients’ ability to communicate effectively, process and retain information, plan and make decisions, or independently manage daily activities [1–4].

These impairments also have direct implications for patients’ interactions with the healthcare system. Reduced cognitive clarity, communicative capacity, or physical function may hinder patients’ ability to actively engage with healthcare professionals (HCPs) and participate in care‐related decision‐making [1–4]. Consequently, patients with ABI or MBT frequently become dependent on relatives for emotional support, practical assistance, coordination of care, and navigation of clinical encounters [5–10]. This growing dependency often prompts relatives to assume new or expanded caregiving responsibilities, such as advocating on behalf of the patient, monitoring symptoms, supporting decision‐making, and serving as intermediaries in communication with HCPs. These evolving roles underscore the importance of relatives’ involvement in clinical care.

Despite broad recognition of the importance of involving relatives in care, the conceptual foundations of involvement remain insufficiently articulated [11]. Terms such as involvement, participation, collaboration, and shared decision‐making are often used interchangeably, hindering consistent implementation in practice [11–14]. In this paper, we adopt a pragmatic working definition of involvement as the active participation of relatives in the disease trajectory, including the reciprocal exchange of knowledge and information among relatives, patients, and HCPs [11, 13]. The purpose is not to resolve theoretical debates but to operationalize the concept in a way that facilitates analysis of how involvement unfolds in everyday clinical practice.

Evidence consistently shows that involvement of relatives is associated with improved psychological well‐being, enhanced patient safety, and better care experiences [15–17]. Family‐centred care interventions across paediatric, neonatal, and critical care settings have demonstrated benefits such as reduced complications, shorter hospital stays, and increased satisfaction [18, 19]. Nurses play a key role in facilitating such involvement due to their continuous presence and close contact with patients and families [20, 21]. Nevertheless, involvement remains a complex, multifaceted process shaped by organizational structures, interpersonal relationships, and the diverse needs and capacities of relatives [22, 23].

Studies focusing on relatives of patients with ABI and MBT consistently report unmet needs related to communication, information, and emotional support [5–10]. These needs are intensified by disease‐specific factors: patients with ABI may experience fluctuating or impaired cognitive capacity, while MBT often involves rapid disease progression and uncertain prognosis. Consequently, relatives frequently assume advocacy roles, support decision‐making, and act as intermediaries in communication with HCPs [24, 25]. These characteristics make ABI and MBT clinically relevant contexts for examining how involvement of relatives is enacted and negotiated in practice. However, little is known about how involvement unfolds in real‐time clinical encounters.

To the best of our knowledge, the involvement of relatives of patients with ABI or MBT has not previously been examined through direct observation in clinical practice. Therefore, this study aims to explore how the involvement of relatives of patients with either an ABI or an MBT unfolds in a hospital ward setting. Particular attention is given to the contextual conditions that shape this involvement, the interpersonal dynamics between relatives and HCPs, and the communication practices that characterize their interactions. Through nonparticipant observational fieldwork, the study seeks to provide an in‐depth understanding of how relative involvement is enacted, negotiated, and challenged in everyday clinical practice.

## 2. Design

This study employed a qualitative observational design informed by Spradley’s ethnographic method [26], which offers a systematic framework for understanding social interaction in its natural context. Spradley’s approach supported an analytical focus on both the broader organizational and cultural conditions surrounding care encounters and the fine‐grained interactional practices unfolding between nurses, patients, and relatives [26]. Introducing this framework at the outset provides transparency regarding the methodological logic that shaped data collection and interpretation.

The study is situated within an interpretivist qualitative tradition, aiming to understand how involvement practices and relational dynamics are constructed and enacted in context rather than to seek a single objective truth. Within an interpretivist orientation, references to neutrality and the researcher’s role reflect a reflexive stance that highlights, rather than conceals, how the researcher’s perspective contributes to the interpretive process. This approach promotes transparency in how understandings are generated. Spradley’s ethnographic lens aligns with this epistemological position by emphasizing situated meaning‐making and the contextual shaping of interaction [26].

A nonparticipant observational stance was chosen to ensure that naturally occurring communication patterns and relational dynamics were captured with minimal intrusion [26]. Although all observers had professional backgrounds in nursing, none participated in clinical tasks during observations. Maintaining a clear and stable observer role reduced the risk of influencing interactions, minimized role confusion for staff, patients, and relatives, and ensured methodological coherence with the aim of examining involvement as it unfolded in everyday clinical practice.

### 2.1. Participants and Selection

A consecutive sampling strategy was employed, whereby all consenting relatives present on the wards during the observation period were included and observed in both formal and informal contexts. This strategy was chosen because consecutive sampling is well suited to dynamic clinical environments, allowing systematic inclusion of all eligible individuals as they appear and ensuring that data reflect naturally occurring interactions relevant to the study aim [27].

When multiple relatives were present, the individual designated by the patient as the primary relative was approached. If the patient was unable to make this decision, the nearest relative listed in the medical record typically a spouse was included.

To explore the interactions between nurses and relatives, the study focused primarily on relatives, nurses, and a supervised nursing student involved in direct patient care. Nurses were eligible if they were employed in direct patient care and routinely interacted with relatives, and all levels of seniority were included. Direct patient care was defined as clinical tasks requiring physical proximity to the patient and active professional responsibility for their treatment or daily care. This included activities such as assessment and monitoring of neurological status, medication administration, personal care tasks, mobilization, and participation in treatment‐related conversations at the bedside.

Involvement of relatives was operationalized as any purposeful interaction between nurses (or the supervised student nurse) and relatives regarding the patient’s condition, care trajectory, or daily needs. This encompassed information‐giving, emotional or practical support, shared decision‐making discussions, and responses to relatives’ questions or concerns during both formal and informal encounters.

Nurses were therefore eligible for inclusion if their role entailed routine execution of these forms of direct patient care and regular engagement with relatives in at least one of the above ways during their typical clinical duties.

A nursing student also contributed to observed encounters as part of her routine clinical duties during data collection. The participating student nurse was in their second semester, working under direct supervision in the ward. Encounters involving the student nurse occasionally reflected more task‐oriented and less confident relational engagement; this potential influence was considered during analysis.

Other HCPs were present in some formal meetings; however, due to the study’s analytical focus on interactions between nurses, the student nurse, patients, and relatives, only demographic data for these three groups were collected.

### 2.2. Inclusion and Exclusion Criteria

Relatives of patients cared for by participating nurses during the observation period were invited to participate unless they met the following exclusion criteria: being under 18 years of age or unable to provide informed consent. No language requirements were applied, as the observations focused on both verbal and nonverbal interaction, and communication could be interpreted independently of spoken language.

Patients were eligible if they were under the care of the observed nurses during the study period. Although patients were not study participants, they were always asked whether they found it acceptable that their relatives took part in the observations. Formal consent was not required from patients, as the focus of the study was on relatives’ involvement rather than on patient behaviour.

### 2.3. Study Setting

Observations were carried out across four hospital sites in Denmark, comprising three departments and seven units admitting adult patients with either ABI or MBT. One site was a neurosurgical department that included a semi‐intensive unit and a surgical ward treating a broad range of brain‐related surgical conditions, including MBT such as gliomas, as well as aneurysms and vascular malformations. This site also comprised a neurological ward responsible for the treatment of conditions such as cerebral infarction and intracerebral haemorrhage. Another site was an inpatient neurorehabilitation centre admitting patients recovering from moderate to severe stroke or traumatic brain injury. Additional observations were conducted at an acute neurosurgical unit and a neurological intermediate care unit, both of which manage adults with acute or fluctuating neurological conditions requiring close monitoring.

Across these varied settings, observations were conducted in both formal and informal contexts. Formal observations included scheduled meetings involving the patient, relatives, and HCPs with a predefined purpose (e.g., treatment planning, discharge discussions, or medical briefings). Informal observations were carried out during spontaneous, everyday situations arising naturally during the patient’s hospital stay, such as bedside conversations, routine care activities, and ad hoc information exchanges.

This combination of diverse clinical environments and observation types provides a richly contextualized dataset and enables later data excerpts to be clearly linked to their respective units and settings.

### 2.4. Data Collection

#### 2.4.1. Nonparticipant Observation

Data were collected through nonparticipant observations guided by an observation framework based on Spradley’s nine descriptive dimensions (space, actors, activities, objects, acts, events, time, goals, and feelings). During each session, the researcher took handwritten field notes that were later transferred into a structured protocol to support consistent organization of the data. These notes contained detailed and neutral descriptions of what relatives and HCPs said and did, together with contextual information about the physical environment, participants’ positioning and bodily conduct, and the atmosphere of the encounter. Each transcript also included preliminary interpretations, analytic reflections, and self‐reflexive considerations regarding the researcher’s positioning and potential influence on the situation.

Inspired by Spradley’s ethnographic method, both grand tour and mini tour observations were employed to structure the fieldwork [26]. Grand tour observations were used to map the broader physical and organizational context, such as the spatial layout of the units, the rhythm of daily clinical routines, and the types of interactions unfolding across different locations. These observations provided insight into how environmental and organizational conditions shaped opportunities for relatives’ presence and involvement. In contrast, mini tour observations focused more narrowly on specific actions and communicative practices, including how nurses and other HCPs acknowledged, engaged with, or directed information toward relatives in concrete care situations. Combining grand and mini tours enabled a layered understanding of both macro‐level organizational conditions and micro‐level relational dynamics and proved particularly effective for examining how relative involvement unfolded in real time.

Observations were conducted across four Danish hospital sites between 6 February and 23 March 2023. In total, 19 observation sessions were completed, yielding 12 h and 40 min of observational material. Individual sessions ranged from 3 min to 2 h and 35 min, with an average duration of 40 min. All observations took place during daytime hours. Table 1 provides an overview of the study sites, associated units, condition focus, number of observation sessions conducted at each location, and the responsible observers.

**TABLE 1:** Overview of study sites, units, number of observation sessions, and observers.

Study site	Unit/department	Condition focus (ABI/MBT/stroke)	Number of observation sessions	Observer^a^
Specialised neurorehabilitation centre A	Semi‐intensive neurorehabilitation ward	ABI	1	PSS
	Neurorehabilitation ward	ABI	4	PSS

Specialised neurorehabilitation centre B	Neurorehabilitation unit A	ABI	1	LA
	Neurorehabilitation unit B	ABI	2	LA

Neurosurgery department	Neurosurgical ward	MBT	4	IP
	Neuro intermediate care unit	ABI	4	ML

Thrombolysis unit	Acute stroke/thrombolysis unit	ABI	3	RG

^a^Initials refer to the researchers who conducted the observational sessions and correspond to the authors of the article.

The observation team consisted of five researchers with nursing backgrounds and varying levels of qualitative research experience; consistency in data collection was supported through ongoing team dialogue and reflexive discussions. To reduce potential influence related to the researchers’ presence and positionality, observations were conducted in settings where the researchers were not employed, and they wore their own clothing and introduced themselves clearly as researchers. Following each session, demographic data were collected for the observed nurse, nursing student, the participating relatives, and the patient under their care.

A typical observation day began with the observer arriving during daytime hours and briefly greeting the ward staff. Once a relevant situation involving a nurse, a patient, and one or more relatives was identified, the observer positioned themselves at the edge of the care environment, such as near a wall or seated slightly apart to avoid influencing the ongoing activities. Interactions were followed as they naturally unfolded, with systematic attention to spatial arrangements, verbal and nonverbal communication, the sequence of activities, and the emotional tone of the encounter. Handwritten field notes were taken continuously, focusing on descriptive accounts rather than evaluative judgements and documenting how relatives and HCPs oriented to one another, exchanged information, and navigated the physical environment.

Immediately after the session, the observer withdrew to a quiet space to expand and clarify the notes while the situation was still fresh. These were then transferred into the structured protocol, where descriptive observations were supplemented with initial analytic thoughts and reflexive considerations regarding the observer’s presence and potential influence. This routine was applied consistently across all sites and sessions, supporting comparability of observations and reducing the risk of selective attention or retrospective reinterpretation.

### 2.5. Data Analysis

The field notes were analysed using a qualitative content analysis inspired by Graneheim and Lundman [28]. This approach allowed for a systematic interpretation of both the manifest content, that is, the visible and descriptive elements of communication and interaction, and the latent content, representing the underlying meaning and interpretive patterns across the dataset. Initially, the analysis focused on the manifest level, aiming to stay close to the empirical material. Observation transcripts were read repeatedly by the first, second, and last authors to gain a holistic understanding of the data. Meaning units were identified and extracted and then condensed and assigned descriptive codes. These codes were sorted into categories, which represented recurrent and observable features of how relative involvement unfolded in the clinical context (Table 2). Through collaborative discussions, the researchers developed and refined three descriptive categories that captured central patterns in the material. In a second interpretive step, the researchers conducted a latent analysis, aiming to capture the underlying meanings and dynamics embedded in the data. This analytic process involved identifying cross‐cutting patterns and connections between categories and reflecting on how relative involvement was shaped by contextual, communicative, and relational dynamics. The outcome of this latent analysis was formulated as a synthesizing, overarching theme, illustrating a continuum of involvement ranging from absence to active partnership. Throughout the analytic process, the authors engaged in reflexive discussions to ensure credibility and transparency, and disagreements were resolved through consensus.

**TABLE 2:** Example of codes, categories, and theme.

Context (Obs #/setting)	Meaning unit (verbatim excerpt)	Condensed meaning unit	Code	Category	Contribution to the overarching theme
Obs 1: admission to ward (rehab. unit)	“The nurse approaches the wife with various papers and informs her about the care pathway conversation, obtains consent to travel by bus and explains that brochures are no longer handed out, but that the information can be found on the website. The wife responds somewhat hesitantly with ‘okay’.”	Nurse provides multiple items of information in a one‐way manner; wife responds hesitantly.	Unidirectional information transfer	Communicative practice. Conditions for understanding, participation, and recognition	It shows how monologic communication positions relatives as recipients, not participants. This interaction sits at the low‐involvement end of the continuum and feeds the theme by illustrating “absence‐like” presence (physically present, interactionally peripheral).
Obs 6: patient room (neuro intermediate care unit)	“The patient’s partner enters and greets the nursing student. The student does not look at the relative and does not respond and then leaves the room.”	Relative’s attempt to engage is not acknowledged; no verbal or nonverbal uptake.	Nonrecognition/missed greeting	Communicative practice. Conditions for understanding, participation, and recognition	It demonstrates interactional nonrecognition, reinforcing involvement as absent. It strengthens the leftmost anchor of the theme’s continuum: relatives can be present yet invisible when communication fails.
Obs 12: bedside, routine care (rehab. unit)	“The nurse places a lip balm and fresh water on the bedside table and points it out to the wife, suggesting that she help. When the nurse has finished her tasks, she leaves the room.”	Implicit invitation for practical help without dialogue about role.	Tacit task delegation	Relational positioning between recognition and distance	It shows mid‐continuum involvement: relatives are drawn into practical support but remain outside planning/decisions. It clarifies how relational positioning shifts involvement from absence to helper, informing the theme’s graded movement.

### 2.6. Ethics

Approval from the Danish data protection agency was obtained, and the study was conducted according to the principles of the Declaration of Helsinki 29 (World Medical Association Declaration of Helsinki: ethical principles for medical research involving human subjects, 2013). Written and verbal information was given before obtaining informed consent to participate in the study. Participants were informed of the study aim and voluntary nature of the study and that withdrawal from the study was possible at any time with no implications for future treatment or rehabilitation. Data were stored in a secure folder according to the data protection rules. Pseudonyms were assigned to all participants.

## 3. Findings

A total of 13 relatives participated in the observations. The group represented a wide range of relational roles, enabling examination of diverse forms of involvement. Their mean age was 65.1 years (Table 3) and they included spouses or partners, siblings, parents, and adult children.

**TABLE 3:** Demographic characteristics of study participants.

Variable	Relatives (*n* = 13)	Nurses (*n* = 14)	Nursing student (*n* = 1)	Patients (*n* = 12)
Age, mean ± SD (years)	65 ± 6.9	38.1 ± 11.8	19	65.0 ± 17.9
Relation to patient				
Spouse/partner	7			
Sibling	2			
Parent/child	2			
Unknown	2			
Professional experience (years)				
Years since graduation		10.4 ± 9.3	0	
Years in current department		6.7 ± 6.4	0	
Clinical diagnosis/condition				
Suspected stroke				3
Stroke (including ischaemic stroke and intracerebral haemorrhage)				4
Subarachnoid haemorrhage				1
Colloid cyst				1
Observation for brain tumour				1
Not reported				2

Observational data were also generated from 14 nurses of varying levels of clinical experience, as well as one nursing student (Table 3). Nurses’ average age was 38.1 years, and they had been registered nurses for an average of 10.4 years.

**FIGURE 1:**
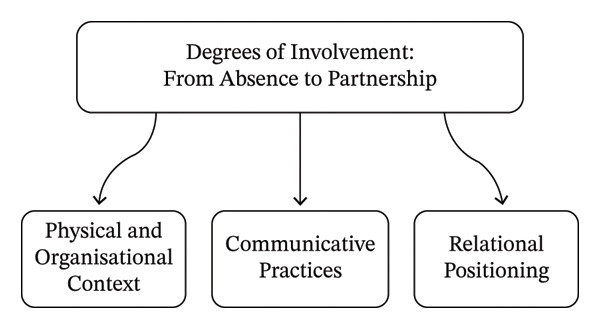
Theme and categories. Figure 1 is generated with the assistance of AI (Microsoft Copilot).

Based on the analysis of 19 observed occasions, one overarching theme, “Degrees of Involvement: From Absence to Partnership,” was identified, together with three interrelated categories: Physical and Organizational Context: Structural Conditions for Involvement; Communicative Practices: Conditions for Understanding, Participation, and Recognition; and Relational Positioning: Between Recognition and Distance (Figure 1). Together, these illustrate how nurses shape whether, when, and how relatives are acknowledged and engaged in the care process. They also demonstrate that involvement is not static but unfolds along a continuum influenced by environmental, communicative, and relational factors.

### 3.1. Theme: Degrees of Involvement: From Absence to Partnership

Across the analysis, involvement emerged not as a fixed state but as a continuum ranging from complete absence to active partnership. This overarching theme captures how relatives’ participation varied in form and intensity depending on contextual conditions, communicative practices, and relational positioning. The continuum became visible as relatives shifted between roles as bystanders, practical helpers, and engaged participants. In some situations, relatives were physically present yet unacknowledged, standing quietly at the margins of clinical activity. In other situations, they were drawn into isolated tasks without being connected to the broader care plan. At the opposite end of the continuum were structured meetings where relatives’ perspectives were actively elicited, validated, and integrated into decision‐making.

The overarching theme shows that involvement is shaped not only by relatives’ willingness or availability but also by the organizational structures, communicative norms, and relational climate of care settings. It highlights the potential for more systematic support to move relatives from passive presence toward meaningful partnership when appropriately aligning their contributions with the patient’s evolving needs.

Category 1: Physical and Organizational Context: Structural Conditions for Involvement: this category captures how spatial arrangements, clinical rhythms, and organizational routines shape what forms of involvement are possible for relatives. These contextual conditions do not merely “set the scene,” they actively structure where relatives are positioned on the continuum of involvement, from noninvolvement to active, facilitated involvement.

In acute settings, the urgency of life‐saving procedures inherently limits relational or communicative involvement. The following excerpt represents a situation close to the noninvolvement end of the continuum:

*Fieldnote, Observation 19 (February 23). “*The patient arrives by ambulance following a referral from their general practitioner due to suspected stroke. She is received in the anteroom of the CT scanner. Her spouse accompanies her and appears visibly frightened. He (the spouse) is informed that the process will move quickly and is asked to wait outside the room. The observer follows him and sits beside him. He is neither given the opportunity to speak with his wife nor provided with any further information. The spouse appears deeply distressed, crying quietly.*”*

Here, the physical layout (anteroom, restricted area), the pace of stroke diagnostics, and professional routines collectively produce a situation where the spouse is physically removed and informationally excluded. His distress is not incidental; it is a direct effect of the structural conditions. This is not a relational decision made by the staff but an organizational necessity that nonetheless has relational consequences.

In contrast, the rehabilitation settings offered more stable opportunities for relatives to remain present throughout the day. However, the data show that presence does not equate to involvement. Often, relatives are physically close but only peripherally engaged, with ambiguity about their role. Observation 12 sits in this “middle” area of the continuum.

*Fieldnote, Observation 12 (March 2023).* “The nurse returns. The wife is standing next to the patient’s bed. The nurse encourages the patient to drink one of the two glasses of potassium, and then she administers insulin. The wife asks why the patient needs to drink potassium and receive insulin now. The nurse explains the reason for the potassium and insulin.”

“The nurse places a lip balm and some fresh water on the patient’s bedside table and points it out to the wife, suggesting she help with this. When the nurse has finished her tasks, she leaves the room.”

Here, the wife is present and even initiates dialogue, but her involvement is fragile and situational. The nurse’s brief explanation and subtle suggestion to assist with basic care create a minimal and task‐oriented form of involvement, but no explicit invitation to participate meaningfully beyond that moment. The spatial arrangement (wife standing at bedside while nurse controls the task sequence) reinforces a passive, waiting posture.

This excerpt illustrates a pattern across the dataset: when involvement is not structurally embedded, it appears only as small, discretionary gestures from staff rather than as a planned or sustained practice.

Category 2: Communicative Practices: Conditions for Understanding, Participation, and Recognition: relatives’ ability to participate was closely linked to communicative patterns. Across the observations, a distinction emerged between monologues and dialogic interactions. In monologic encounters, HCPs conveyed information without inviting response. Dialogic practices, in contrast, actively included relatives through questions, attentive listening, and acknowledgement of their knowledge.

In both acute and nonacute environments, relatives were often physically present but communicatively excluded. The following excerpt illustrates a near‐complete absence of recognition. It sits at the noninvolvement end of the continuum:

*Fieldnote, Observation 6 (March 2023). “*The nursing student enters the patient’s room to inform her that her peripheral venous catheter (PVC) can be removed. The patient had previously mentioned that it was causing discomfort and, according to her, had not been used for anything since she arrived in the ward the day before. The patient’s partner enters the room. He walks over to the patient, kisses her twice, and then goes to an armchair where he takes off his jacket. He greets the nursing student. He sits down in the armchair. The student does not look at the relative and does not respond the greeting. The nursing student then leaves the room.*”*

Relatives may attempt to engage in clinical interactions through gestures or greetings yet remain unacknowledged. The absence of verbal or nonverbal response, as observed in the interaction with a nursing student, reflects a lack of mutual engagement. This lack of recognition creates barriers to shared understanding and may lead relatives to feel invisible, influencing how they perceive their role and presence in the care setting. In contrast, structured meetings demonstrated a markedly different communication climate. These encounters included relatives through deliberate turn‐taking, clarification, and recognition of relational knowledge. The following example sits toward the meaningful involvement end of the continuum:

*Fieldnote, Observation 15 (March 2023).* “The spouse expresses her surprise at the patient’s significant progress, exemplifying this by explaining that, despite ongoing challenges with letter recognition, he is now able to forward emails to her. She emphasizes her strong wish for the patient to remain in the rehabilitation unit for as long as possible in order to benefit fully from the rehabilitation process, noting that weekend visits are appreciated. The physiotherapist underscores that weekends should include not only continued training but also ample time for rest and recovery. The occupational therapist adds, “training is also about engaging in conversations and being able to prioritize where to spend one’s energy.” The spouse refers to situations when the couple receives visits from friends, which has previously caused severe fatigue for the patient, prompting them to adjust their activities accordingly. The occupational therapist acknowledges this and affirms that they are working purposefully with energy management in line with the patient’s and spouse’s goals.*”*

The fieldnote illustrates a communicative environment that contrasts with the monologic and unreciprocated exchanges often observed in informal ward encounters. In this meeting, the spouse shares her observations of the patient’s progress and articulates her wish for continued rehabilitation. The HCPs respond directly to her contributions, elaborating on the balance between training, rest, and energy management, and they acknowledge the relevance of her experiences to the patient’s daily functioning.

The tone, eye contact, and turn‐taking in this setting demonstrate a more deliberate and inclusive communication style. The HCPs pause to ensure understanding, use accessible language, and recognize the emotional and experiential dimensions brought forward by the spouse. Such dialogic practices enable relatives to move from passive recipients to active partners in care planning.

By contrast, informal encounters often lacked verbal or nonverbal acknowledgement, leaving relatives uncertain about their role. This inconsistency highlights the need for more relationally attuned communication practices to support meaningful and sustained involvement across clinical contexts.

Category 3: Relational Positioning: Between Recognition and Distance: relatives were positioned not only through the physical layout of the room but also relationally through verbal and nonverbal cues. Their role often remained unclear, fluctuating between helper, bystander, and informal expert. These shifting positions were shaped by how nurses oriented themselves towards relatives during interaction.

In several encounters, relatives adopted a helper role in response to subtle, often unspoken expectations:

*Fieldnote*, *Observation 3 (February 2023).* “The spouse asks where he can find some cutlery, and the nurse offers to show him. The nurse invites the spouse to come along and see where the cutlery is located. The nurse leaves the room, and the spouse follows a few steps behind her down the hallway.”

Such micro‐involvements were practical and task‐oriented, offering participation without granting influence or voice in clinical decisions.

In other situations, relatives’ presence remained peripheral until their knowledge was required. In Observation 1 (February 2023), the spouse stands quietly in the corner while the nurse addresses the patient. Only when the nurse needs clarification about the type of wheelchair does she turn to the spouse, who then provides detailed information. This interaction exemplifies reactive involvement: relatives are drawn in only when their expertise becomes functionally relevant, reinforcing a dynamic where HCPs determine the timing and scope of their participation. *Fieldnote, Observation 1 (February 2023). “*The physiotherapist begins by welcoming the patient and informing him about what will happen during the day. Meanwhile, the nurse greets the patient’s wife. She is still standing in the corner diagonally across from the bed, holding her coat and bags, silently observing. The nurse then returns to the bedside (with her back to the wife) and continues talking to the patient about the course of the day. The nurse asks which wheelchair the patient uses, to which the patient responds uncertainly. The nurse then asks the wife about the wheelchair, and she adds information about which model (name of the patient) started with, when, and which model he has transitioned to as a result of increased mobility.*”*

This observation illustrates how relatives often remain positioned at the margins until their knowledge becomes operationally relevant. While the physiotherapist welcomes the patient and explains the plan for the day, the patient’s wife stands silently in the corner, holding her coat and observing. Although the nurse briefly greets her, she then turns her back and continues speaking to the patient. It is only when the nurse requires clarification about the patient’s wheelchair that she addresses the wife, who provides detailed information about the model and the patient’s recent transition to a different type as his mobility improved.

This interaction exemplifies a pattern in which involvement is reactive rather than proactively supported. Relatives move from the periphery into the interaction only when their knowledge fills a gap, sustaining a relational asymmetry in which HCPs determine both the timing and the scope of their participation.

In contrast, structured meetings disrupted this dynamic by offering a defined communicative space where relatives were invited to contribute from the outset. When involvement was pre‐planned, relatives shifted from reactive informants to co‐creative partners whose concerns, insights, and emotions were recognized as legitimate contributions to the care process.

*Fieldnote, Observation 5 (February 2023).* “The relative expresses his belief that strength training is important for the patient and suggests that the patient could practice walking with a cane. The nurse responds that the most important thing right now is to train the patient’s balance, so the patient can use the Sara Stedy (a sit‐to‐stand patient transfer aid). Strength training will come afterwards. The occupational therapist adds that strength training is a good idea, but that the timing is not right at the moment. The occupational therapist elaborates that it is best to use the training window to focus on improving balance and strength on the affected side. If the patient were to train with a cane now, the brain would automatically focus on the healthy side. Both the patient and the relative express that this explanation makes good sense.”

Relational positioning was also shaped by nonverbal signals such as whether relatives were greeted, offered a chair, or included in eye contact. Being left standing in the background conveyed distance or marginality, whereas small gestures of recognition generated a sense of belonging within the interactional space. These embodied cues often communicated inclusion or exclusion more powerfully than spoken words.

## 4. Discussion

This study provides new insights into how relatives’ involvement unfolds in neurological and neurosurgical hospital settings. The observational data show that involvement is highly context‐dependent and varies along a continuum ranging from absence to active partnership. Although the background references all HCPs, the empirical material is derived primarily from nurses, reflecting their continuous presence in everyday care. This focus also represents a limitation, as interactions with other professional groups were not systematically observed.

In acute clinical scenarios, such as the assessment of patients for suspected stroke or thrombolysis, relatives were typically involved only to the extent of providing information. These findings align with prior literature, which suggests that in life‐threatening situations, relatives are rarely included in shared decision‐making due to time constraints, clinical urgency, and patient incapacity [[30, 31]]. However, even in such circumstances, clear, timely, and empathetic communication from HCPs can constitute a meaningful form of participation [21].

In less acute and more informal contexts, the degree of involvement varied considerably. Observations ranged from relatives being passive bystanders to active contributors in formal meetings. Structured interactions, such as multidisciplinary planning sessions, provided a clear framework that enabled professionals to invite relatives into dialogue, acknowledge their perspectives, and integrate their knowledge into care planning. These findings mirror previous studies conducted in intensive care and rehabilitation settings, where structured communication has been shown to facilitate trust and a sense of partnership, even in the absence of full decision‐making authority [30, 32].

A key finding is that relatives were often physically present but rarely acknowledged as active participants in the care process. Their involvement was typically ad hoc and often depended on their own initiative. These findings align with previous research showing that although relatives frequently express a strong desire to participate in care and decision‐making, their involvement is often insufficiently facilitated or proactively supported by HCPs [19, 30, 33, 34]. Barriers to systematic involvement may include competing clinical demands, limited experience among HCPs in collaborating with families, and uncertainty about when and how relatives should be engaged [35]. Moreover, involvement was frequently influenced by individual HCPs’ attitudes rather than institutional practices, resulting in significant variation and, at times, exclusion [30, 31, 35].

The present findings also support prior evidence that relatives are not a homogeneous group; rather, they bring varying resources, needs, and capacities for involvement [36]. Observations highlighted that even relatives who appeared passive or hesitant could engage meaningfully when professionals created a safe and appreciative environment for dialogue. This suggests that successful involvement is not merely about the relatives’ initiative but also about the ability of HCPs to facilitate inclusive practices. Previous studies have described archetypical roles that relatives assume, such as the “warrior,” the “observer,” or the “hesitant” [36], but our data suggest these roles are fluid and shaped by both context and lack of interaction.

Overall, the study underscores that involvement is a dynamic process shaped by clinical setting, communication style, and relational positioning. Without systematic frameworks for recognizing relatives as part of the care continuum, involvement tends to occur only in response to immediate clinical needs. To foster meaningful participation, interventions are needed that support nurses in identifying relatives’ preferences, offering clear invitations to participate, and creating consistent structures for inclusion. Such approaches require organizational backing, reflexive practice, and relational sensitivity, ensuring that involvement aligns with both the patient’s condition and the relative’s capacity and wishes.

## 5. Methodological Considerations

This study has several methodological limitations that should be considered when interpreting the findings. First, observations were conducted during daytime hours, which may have limited insight into involvement occurring during evenings, nights, or weekends. Daytime routines often involve higher activity levels and tightly scheduled clinical tasks, potentially reducing opportunities for engagement with relatives and shaping the interactions captured. Second, although observations spanned the patient pathway from acute assessment to rehabilitation, capturing hyperacute interactions was challenging due to clinical urgency and rapid decision‐making. Some nuances of involvement in these critical moments may therefore not have been fully observed.

All researchers involved in data collection had professional backgrounds in nursing, which may have shaped how interactions were interpreted and documented. While this provided contextual sensitivity and facilitated access, it also introduced the possibility of insider perspective. Beyond influencing data collection, the researchers’ nursing backgrounds also shaped the interpretation of latent content during analysis. Their clinical experience provided nuanced insight into implicit relational cues, power dynamics, and tacit aspects of communication that may not be immediately visible in surface‐level descriptions. At the same time, this insider perspective carried a risk of normalizing certain practices or overlooking alternative interpretations.

To address this, the research team engaged in continuous reflexive dialogue, comparing preliminary interpretations, challenging assumptions, and revisiting field notes to ensure that latent meanings were grounded in the empirical material rather than disciplinary preconceptions. The researchers focused on articulating their positionalities and examining how their professional backgrounds and physical presence informed the production of knowledge. Reflexive notes were used to trace these interpretive processes and document how understandings developed over time.

The presence of observers may have influenced nurses’ behaviour, potentially increasing their attentiveness towards relatives. The researchers acknowledged their presence as part of the relational context and sought to position themselves in ways that supported openness while minimising disruption to clinical work. Variability in observational style and interpretation among the research team could also have influenced data consistency. Additionally, the presence of a supervised student nurse in the observed environments introduced a training‐related dynamic. Her experience level may have shaped communicative patterns, and this was considered in the analysis. The findings highlight the need for future recommendations to address how student nurses are supported, supervised, and educated in involving relatives in care.

Despite these limitations, nonparticipant observation was well suited to capturing relational and contextual aspects of involvement as they unfolded in everyday clinical practice. Conducting observations across multiple sites and phases of care strengthened the contextual grounding of the findings and provided insight into how involvement varied across different organizational settings.

## 6. Conclusion

This study has illuminated how the involvement of relatives in neurological and neurosurgical hospital settings unfolds as a situated and often unpredictable process, shaped by varying degrees of presence, communication, and recognition. While relatives are frequently physically present, they are often marginalized in terms of relational and communicative engagement. Involvement rarely appears as a systematic and integrated component of care, but rather emerges situationally, dependent on context, clinical routines, and the initiative of individual nurses. Through the identification of one overarching theme supported by three interrelated categories, the analysis highlights how structural, communicative, and relational conditions influence whether, how, and to what extent relatives are included in patient care. The findings underscore the need for more consistent, differentiated, and proactive approaches to involvement, in which nurses take active responsibility for creating spaces and opportunities for meaningful participation. Particularly during transitions from acute to more stable phases of care, opportunities arise to reframe the role of relatives from passive bystanders to active partners. This study contributes important insights for the development of future interventions and clinical practices aimed at promoting meaningful, context‐sensitive involvement of relatives. Such efforts require both organizational support and ongoing professional reflection on the role relatives can and should play, depending on their preferences, capacities, and the patient’s evolving needs.

### 6.1. Implications for Practice

Ensuring equitable and consistent involvement requires clinical teams to establish clear routines for initiating dialogue with relatives, particularly during key transitions in the patient trajectory and to create physical and communicative spaces that support participation, including settings suited for sensitive conversations and relational exchange. It also involves recognizing relatives as knowledgeable partners, whose insights can strengthen continuity, safety, and person‐centred care, and ensuring that nurses receive organizational support and training to develop the relational competencies needed to navigate complex family dynamics. Embedding these practices into everyday care can shift relative involvement from a reactive response to an integrated, relationally grounded component of clinical practice, acknowledging relatives not simply as visitors but as integral contributors to the patient’s pathway. Given the importance of relational competence in nursing, student nurses require structured and supervised opportunities to practice and develop the skills necessary to involve relatives in meaningful and supportive ways.

## Funding

This study was funded by The Danish Health Confederation and Danish Regions, 2657.

## Conflicts of Interest

The authors declare no conflicts of interest.

## Data Availability

The data that support the findings of this study are available from the corresponding author upon reasonable request.

## References

[1] Goldman L. , Siddiqui E. M. , Khan A. et al., Understanding Acquired Brain Injury: a Review, Biomedicines. (2022) 10, no. 9, 10.3390/biomedicines10092167. PMC9496189 36140268

[2] Lanzillo B. , Piscosquito G. , Marcuccio L. , Lanzillo A. , and Vitale D. F. , Prognosis of Severe Acquired Brain Injury: Short and long-term Outcome Determinants and Their Potential Clinical Relevance After Rehabilitation. A Comprehensive Approach to Analyze Cohort Studies, PLoS One. (2019) 14, no. 9, 10.1371/journal.pone.0216507, 2-s2.0-85072663804. PMC6762165 31557186

[3] Mar J. , Arrospide A. , Begiristain J. M. , Larrañaga I. , Elosegui E. , and Oliva-Moreno J. , The Impact of Acquired Brain Damage in Terms of Epidemiology, Economics and Loss in Quality of Life, BMC Neurology. (2011) 11, no. 1, 10.1186/1471-2377-11-46, 2-s2.0-79955082101. PMC3098775 21496356

[4] Molassiotis A. , Wilson B. , Brunton L. , Chaudhary H. , Gattamaneni R. , and McBain C. , Symptom Experience in Patients with Primary Brain Tumours: a Longitudinal Exploratory Study, European Journal of Oncology Nursing. (2010) 14, no. 5, 410–416, 10.1016/j.ejon.2010.03.001, 2-s2.0-78049453412. 20363189

[5] Bayen E. , Laigle-Donadey F. , Prouté M. , Hoang-Xuan K. , Joël M. E. , and Delattre J. Y. , The Multidimensional Burden of Informal Caregivers in Primary Malignant Brain Tumor, Supportive Care in Cancer. (2017) 25, no. 1, 245–253, 10.1007/s00520-016-3397-6, 2-s2.0-84987667323. 27624465

[6] Lien A. W. and Rohde G. , Coping in the Role as next of Kin of a Person with a Brain Tumour: a Qualitative Metasynthesis, BMJ Open. (2022) 12, no. 9, 10.1136/bmjopen-2021-052872. PMC9445781 36691153

[7] Coco K. , Tossavainen K. , Jaaskelainen J. E. , and Turunen H. , Support for Traumatic Brain Injury Patients’ Family Members in Neurosurgical Nursing: a Systematic Review, Journal of Neuroscience Nursing. (2011) 43, no. 6, 337–348, 10.1097/jnn.0b013e318234ea0b, 2-s2.0-84856404210. 22089411

[8] Guldager R. , Nordentoft S. , Poulsen I. , Aadal L. , and Loft M. I. , Wants and Needs for Involvement Experienced by Relatives of Patients with an Acquired Brain Injury: a Scoping Review, JBI Evidence Synthesis. (2022) 21, no. 5, 886–912, 10.11124/jbies-22-00022. 36729839

[9] Guldager R. , Nordentoft S. , Poulsen I. , Aadal L. , and Loft M. I. , Wants and Needs for Involvement Experienced by Relatives of Patients with a Malignant Brain Tumour: a Scoping Review, JBI Evidence Synthesis. (2022) 2022. 10.11124/JBIES-22-00022 36729839

[10] Piil K. , Laegaard Skovhus S. , Tolver A. , and Jarden M. , Neuro-Oncological Symptoms: a Longitudinal Quantitative Study of Family Function, Perceived Support, and Caregiver Burden, Journal of Family Nursing. (2022) 28, no. 1, 43–56, 10.1177/10748407211029986. 34286624

[11] Sahlsten M. J. , Larsson I. E. , Sjöström B. , and Plos K. A. , An Analysis of the Concept of Patient Participation, Nursing Forum. (2008) 43, no. 1, 2–11, 10.1111/j.1744-6198.2008.00090.x, 2-s2.0-42949097484. 18269439

[12] Guldager R. , Nordentoft S. , Aadal L. , Loft M. I. , and Poulsen I. , Wants and Needs of Relatives’ Involvement in Patient Care: the Same but Different, JBI Evidence Synthesis. (2023) 21, no. 11, 2154–2155, 10.11124/jbies-23-00469. 37935421

[13] Hemle Jerntorp S. , Jakobsson J. , Axelsson M. , Carlson E. , and Aho A. C. , Family Members’ Experience of Involvement in the Patient Care Process on an Interprofessional Training Ward: a Qualitative Interview Study, Journal of Interprofessional Education & Practice. (2025) 39, 10.1016/j.xjep.2025.100742. 39266451

[14] Kvarnström S. , Willumsen E. , Andersson-Gäre B. , and Hedberg B. , How Service Users Perceive the Concept of Participation, Specifically in Interprofessional Practice, British Journal of Social Work. (2011) 42, no. 1, 129–146, 10.1093/bjsw/bcr049, 2-s2.0-84856375521.

[15] Kang J. , Shin D. W. , Choi J. E. et al., Factors Associated with Positive Consequences of Serving as a Family Caregiver for a Terminal Cancer Patient, Psycho-Oncology. (2013) 22, no. 3, 564–571, 10.1002/pon.3033, 2-s2.0-84874738531. 22275212

[16] Mackie B. R. , Marshall A. P. , and Mitchell M. L. , Exploring Family Participation in Patient Care on Acute Care Wards: a mixed-methods Study, International Journal of Nursing Practice. (2021) 27, no. 2, 10.1111/ijn.12881. 32856360

[17] Prior S. J. and Campbell S. , Patient and Family Involvement: a Discussion of Co-Led Redesign of Healthcare Services, J Particip Med. (2018) 10, no. 1, 10.2196/jopm.8957, 2-s2.0-85042118259. PMC7489197 33052119

[18] Aljawad B. , Miraj S. A. , Alameri F. , and Alzayer H. , Family-Centered Care in Neonatal and Pediatric Critical Care Units: a Scoping Review of Interventions, Barriers, and Facilitators, BMC Pediatrics. (2025) 25, no. 1, 10.1186/s12887-025-05620-w. PMC11995472 40223058

[19] Joo Y. , Jang Y. , and Kwon O. Y. , Contents and Effectiveness of patient- and family-centred Care Interventions in Adult Intensive Care Units: a Systematic Review, Nursing in Critical Care. (2024) 29, no. 6, 1290–1302, 10.1111/nicc.13105. 38899600

[20] Cranley L. A. , Lam S. C. , Brennenstuhl S. et al., Nurses’ Attitudes Toward the Importance of Families in Nursing Care: a Multinational Comparative Study, Journal of Family Nursing. (2022) 28, no. 1, 69–82, 10.1177/10748407211042338. 34493109 PMC8814953

[21] Kitson A. , Conroy T. , Kuluski K. , Locock L. , and Lyons R. , *Reclaiming and Redefining the Fundamentals of Care: Nursing’s Response to Meeting Patients*, 2013.

[22] Kiwanuka F. , Sak-Dankosky N. , Alemayehu Y. H. , Nanyonga R. C. , and Kvist T. , The Evidence Base of nurse-led Family Interventions for Improving Family Outcomes in Adult Critical Care Settings: a Mixed Method Systematic Review, International Journal of Nursing Studies. (2022) 125, 10.1016/j.ijnurstu.2021.104100. PMC8560087 34736074

[23] Kiwanuka F. , Shayan S. J. , and Tolulope A. A. , Barriers to Patient and family-centred Care in Adult Intensive Care Units: a Systematic Review, Nursing Open. (2019) 6, no. 3, 676–684, 10.1002/nop2.253, 2-s2.0-85069753266. 31367389 PMC6650666

[24] Clayton J. M. , Hancock K. , Parker S. et al., Sustaining Hope when Communicating with Terminally Ill Patients and Their Families: a Systematic Review, Psycho-Oncology. (2008) 17, no. 7, 641–659, 10.1002/pon.1288, 2-s2.0-48449106403. 18022831

[25] Hudson P. and Payne S. , Family Caregivers and Palliative Care: Current Status and Agenda for the Future, Journal of Palliative Medicine. (2011) 14, no. 7, 864–869, 10.1089/jpm.2010.0413, 2-s2.0-79959749511. 21599532

[26] Spradley J. P. , Participant Observation, 1980, Holt, Rinehart and Winston.

[27] Polit D B. C. , Developing a Sampling Plan’, in Nursing Research, Generating and Assessing Evidence for Nursing Practice, 2018, Lippincott Williams & Wilkins.

[28] Graneheim U. H. and Lundman B. , Qualitative Content Analysis in Nursing Research: Concepts, Procedures and Measures to Achieve Trustworthiness, Nurse Education Today. (2004) 24, no. 2, 105–112, 10.1016/j.nedt.2003.10.001, 2-s2.0-1242331407. 14769454

[29] Medical Association Declaration of Helsinki: Ethical Principles for Medical Research Involving Human Subjects, JAMA. (2013) 310, no. 20, 2191–2194, 10.1001/jama.2013.281053, 2-s2.0-84888610885. 24141714

[30] Guldager R. , Sejr Smedegaard P. , Nordentoft S. , Aadal L. , Ingerslev Loft M. , and Poulsen I. , Facilitators and Barriers of Relatives’ Involvement in Care of Patients with Acquired Brain Injury or Malignant Brain Tumour: Scoping Review, Nursing Open. (2026) 13, no. 1, 10.1002/nop2.70417. PMC12819177 41559742

[31] Wubben N. , van den Boogaard M. , van der Hoeven J. G. , and Zegers M. , Shared decision-making in the ICU from the Perspective of Physicians, Nurses and Patients: a Qualitative Interview Study, BMJ Open. (2021) 11, no. 8, 10.1136/bmjopen-2021-050134. PMC8359489 34380728

[32] Ashana D. C. and Cox C. E. , ICU Communication: ‘State of the Art, Intensive Care Medicine. (2026) 10.1007/s00134-026-08364-y. PMC13277582 41817730

[33] Asadi N. and Salmani F. , The Experiences of the Families of Patients Admitted to the Intensive Care Unit, BMC Nursing. (2024) 23, no. 1, 10.1186/s12912-024-02103-8. PMC11197245 38918819

[34] Xyrichis A. , Fletcher S. , Philippou J. , Brearley S. , Terblanche M. , and Rafferty A. M. , Interventions to Promote Family Member Involvement in Adult Critical Care Settings: a Systematic Review, BMJ Open. (2021) 11, no. 4, 10.1136/bmjopen-2020-042556. PMC8031009 33827833

[35] Paxino J. , Denniston C. , Woodward-Kron R. , and Molloy E. , Communication in Interprofessional Rehabilitation Teams: a Scoping Review, Disability & Rehabilitation. (2022) 44, no. 13, 3253–3269, 10.1080/09638288.2020.1836271. 33096000

[36] Guldager R. , Willis K. , Larsen K. , and Poulsen I. , Relatives’ Strategies in Subacute Brain Injury Rehabilitation: the Warrior, the Observer and the Hesitant, Journal of Clinical Nursing. (2019) 28, no. 1-2, 289–299, 10.1111/jocn.14598, 2-s2.0-85058463925. 29964307

